# Antifungal Resistance, Metabolic Routes as Drug Targets, and New Antifungal Agents: An Overview about Endemic Dimorphic Fungi

**DOI:** 10.1155/2017/9870679

**Published:** 2017-06-13

**Authors:** Juliana Alves Parente-Rocha, Alexandre Melo Bailão, André Correa Amaral, Carlos Pelleschi Taborda, Juliano Domiraci Paccez, Clayton Luiz Borges, Maristela Pereira

**Affiliations:** ^1^Departamento de Bioquímica e Biologia Molecular, Instituto de Ciências Biológicas, Universidade Federal de Goiás, Goiânia, GO, Brazil; ^2^Instituto de Patologia Tropical e Saúde Pública, Universidade Federal de Goiás, Goiânia, GO, Brazil; ^3^Departamento de Microbiologia do Instituto de Ciências Biomédicas e Laboratório de Micologia Médica LIM53/IMTSP, Universidade de São Paulo, São Paulo, SP, Brazil

## Abstract

Diseases caused by fungi can occur in healthy people, but immunocompromised patients are the major risk group for invasive fungal infections. Cases of fungal resistance and the difficulty of treatment make fungal infections a public health problem. This review explores mechanisms used by fungi to promote fungal resistance, such as the mutation or overexpression of drug targets, efflux and degradation systems, and pleiotropic drug responses. Alternative novel drug targets have been investigated; these include metabolic routes used by fungi during infection, such as trehalose and amino acid metabolism and mitochondrial proteins. An overview of new antifungal agents, including nanostructured antifungals, as well as of repositioning approaches is discussed. Studies focusing on the development of vaccines against antifungal diseases have increased in recent years, as these strategies can be applied in combination with antifungal therapy to prevent posttreatment sequelae. Studies focused on the development of a pan-fungal vaccine and antifungal drugs can improve the treatment of immunocompromised patients and reduce treatment costs.

## 1. Introduction

Endemic mycoses as well as some other mycoses include self-limiting cutaneous, subcutaneous, systemic, and disseminated infections. People living in areas of endemic mycosis caused by dimorphic fungi are exposed to and can acquire fungal infections [1–3]. Fungal infections are responsible for morbidity and mortality which are accompanied by high costs to the health system. Antifungal chemotherapy is generally required to treat fungal infections. Despite its modest efficacy against fungal cells and the frequent relapse observed, it is currently the best treatment option. Unfortunately, treatment lasts several months, and the derivation of treatment may last for up to 2 years with sulfonamides, polyenes, azoles, or echinocandins (reviewed in [4]).

Increasing mortality rates caused by fungal diseases, often with the appearance of antifungal-resistant lineages, motivates a scientific race to discover new drug targets. Fungal infection can occur in patients who are immunosuppressed due to organ transplantation, intensive care unit hospitalization, cancer, HIV, surgery, or leukemia as well as in patients who use antibiotics able to modify human microbiota. The number of immunocompromised patients has increased in recent decades and unfortunately continues to grow. Cases of fungal resistance have accompanied this growth [5]. Due to the immunological conditions of patients, the microorganisms grow in an environment where the current therapeutic arsenal is not efficient because the natural immune system is essential to assist in combating infection [6]. The overall inefficiency and high toxicity of current antifungal therapies are also noteworthy [5]. In this context, it is very important to develop a new class of broad-spectrum antifungal agents to circumvent these problems.

## 2. Antifungal Resistance Mechanisms

From a clinical perspective, drug resistance occurs when the appropriate drug therapy is not effective, causing persistence/progression of an infection [7]. However, the molecular mechanisms that lead to antifungal resistance are complex. Fungal cells must commonly adapt to the presence of toxic drugs; the primary molecular survival strategies include (1) mutation of drug targets that reduces its affinity for the drug, (2) overexpression of the targeted protein by modification of the promoter region of the gene, (3) expression of an efflux system, (4) degradation of the drugs, and (5) pleiotropic drug responses [8]. The similarity of the biology of the eukaryotic host and fungal pathogens has led to a drug development focused on the specific characteristics of fungal cells. For example, the fungal cell wall is a specific antifungal drug target. In addition, ergosterol is present in fungal membranes and can be used as a drug target, because it is distinct from the cholesterol present in mammalian cells. Commercial antifungal drugs that act on the cell wall or on ergosterol include azoles, echinocandins, and polyenes.

### 2.1. Fungal Resistance to Azoles

In the last 25 years, several azole drugs (including imidazoles and triazoles) have been used against fungal diseases. These drugs target lanosterol demethylase (cytochrome P450), which has evolved to participate in ergosterol biosynthesis. This enzyme is encoded by the *ERG*11 gene; its inactivation prevents ergosterol production, leading to the accumulation of methylated sterols such as the toxic 14-*α*-methyl-ergosta-8-ene-3,6-diol in the fungal cell [9]. The resistance mechanisms to these drugs are complex. The mutations in the genes *CYP*51A and *CYP*51B (homologous to *ERG*11) are described as reducing the interaction of the drug with the genetic product. The substitutions G54, P216, F219, M220, and G448 have been described as conferring azole resistance [10]. The efflux of azoles has also been reported and is performed by an ATP-binding cassette (ABC) transporter. In *Saccharomyces cerevisiae*, *PDR5* and *PDR15* transporters are overexpressed in azole-resistant strains and are described as conferring azole resistance through efflux [11]. In the last ten years, azole resistance caused by environmental exposure has been studied extensively. This resistance is likely the result of a TR motif (a tandem repeat motif with at least 34-base TR) in the promoter region of *CYP*51A and amino acid mutation, which is well characterized in *Aspergillus fumigatus*, that leads to overexpression of the drug protein target. It is established that this resistance mechanism was developed after exposure of fungal cells to azoles [12]. Recently, the TR34 motif was found associated with the sterol regulatory element-binding protein (SREBP) SrbA and the CCAAT-binding complex (CBC), which is related to repression of sterol synthesis [13]. In this case, the azole tolerance is the result of CBC repression that increases sterol synthesis. In *Cryptococcus neoformans*, the exposure of infected mice to a low dose of fluconazole increases the minimum inhibitory concentration (MIC) of colonies recovered from animals and increases the melanization and capsule size, which are classical virulence factors in this fungus, showing that previous exposure to antifungals leads to different resistance mechanisms in different fungal species [14]. The degradation of azoles is not well characterized, but studies performed with *A. fumigatus* correlate the mitochondrial complex I with metabolism of azoles, causing drug resistances, as mutants of this complex lost azole resistance [15]. Multidrug resistance (MDR) can also lead to azole resistance and includes the phenotype achieved by interactions between transcriptional activators, mediator complexes, and efflux pumps acting as a network in response to several molecules. Azole resistance caused by MDR is described in *S. cerevisiae* and includes the expression of genes regulated by two homologous transcriptional factors *PDR*1 and *PDR*3 [16]. Azole resistance related to the MDR efflux system is also reported in the economically important plant pathogen *Zymoseptoria tritici*, because overexpression of the MgMSF1 transporter was detected in azole-resistant strains [17].

The fluconazole resistance was reported in the dimorphic fungus *Histoplasma capsulatum*, causing treatment failure in HIV patients. In this case, itraconazole was more effective for treatment [18].

### 2.2. Fungal Resistance to Echinocandins

The echinocandins (lipopeptide molecules) are one of few new classes of antifungals to reach the clinic in the last decade. This class includes caspofungin, micafungin, and anidulafungin. These lipopeptides act on fungal cell walls by specific inhibition of 1,3-*β*-D-glucan synthase, which is responsible for the biosynthesis of *β*-1,3-glucan, the key fungal cell wall component [19]. The enzyme consists of three FKS subunits, called FKS1, FKS2, and FKS3 [20]. Resistance is often acquired during therapy by modifying amino acid residues in the FKS1 and FKS2 subunits of *β*-1,3-glucan synthase. [21–23]. The function of the FKS3 subunit remains unclear [23–25].

The mutations in FKS1 and FKS2 that code for catalytic subunit genes can be amino acid substitutions that increase MIC levels accompanied by a dramatic reduction in glucan synthase activity [26–28]. For example, substitutions related to the FKS1 gene in residues Phe641, Pro649, and Arg1361 (*C. albicans* homolog) have been described [29, 30]. For the FKS2 subunit, the substitutions Ser641 and Ser645 strongly reduce enzyme activity, causing a pronounced resistance phenotype [20, 31]. The FKS1 and FKS2 amino acid residue substitutions can vary depending on fungal species but also cause echinocandin resistance [32–34].

*β*-1,3-glucan synthase echinocandin inhibition leads to cell wall defects, which in turn cause cellular stress, and several genes are expressed to adapt to this stress condition. The signaling pathway driven by protein kinase C (PKC) alters the biosynthesis of various carbohydrates related to the cell wall, HOG (high-osmolarity glycerol) [35] and chitin biosynthesis [36, 37]; higher chitin levels are correlated with echinocandin tolerance [38–40]. Altogether, these salvaging pathway phenomena can help the fungus in adaptive cell wall remodeling that enables the cells to survive, even with greater echinocandin doses.

Dimorphic fungi have a natural resistance to echinocandins during the pathogenic phase, but the resistance mechanisms of the *β*-glucan synthase inhibitors are currently unknown [41].

### 2.3. Fungal Resistance to Polyenes

Polyenes are fungicidal and act on the membrane. These molecules are natural fermentation products of *Streptomyces*, and the most used polyene drug is amphotericin B (used in systemic mycoses). For many years, it was believed that polyenes (amphipathic drugs) bind strongly to ergosterol, destroying the proton gradient and allowing leakage of ions [42]. Today, it is known that drug-lipid complexes extract ergosterol from phospholipid in the membrane, depleting ergosterol in the cell [43]. Amphotericin B is refractory to the development of resistance, despite its use for 50 years in clinical treatment. In *Candida tropicalis*, low ergosterol content in the membrane is associated with reduced susceptibility to amphotericin B [44]. Because amphotericin B increases the level of reactive oxygen species (ROS) in fungal cells [45], amphotericin B-resistant *C. tropicalis* produces less ROS and alters mitochondrial activity [46]. In *Aspergillus terreus*, the oxidative damage caused by amphotericin B is more important in causing cell injury than membrane permeation [47].

## 3. Metabolic Routes as Antifungal Drug Targets

In recent decades, the ability of researchers to identify and validate antifungal targets has significantly improved due to improvements in genetic tools for manipulation of fungal pathogens, which lead to a wave of data arising from omics studies and the standardization of animal models for fungal infection. This research progress has begun to allow rational drug design, which creates more efficient antifungal effects with high specific toxicity [48]. In the pipeline for novel investigation of new molecular targets, the following major concerns should be taken into account: (1) the fungicidal target must be essential for fungal survival throughout the infectious process; (2) as mammalian and fungi share basic eukaryotic characteristics, the target or inhibitor must present highly selective toxicity to provide a favorable therapeutic-toxic ratio; and (3) widespread targets among the fungal pathogens must be economically attractive. In addition, the new drug must be safe for use in fragile patients with fungal infections.

### 3.1. Trehalose Metabolism Enzymes as Targets

In the search for new antifungal drugs, the trehalose [*α*-D-glucopyranosyl-(1 → 1)-*α*-D-glucopyranoside] biosynthetic pathway arises as a potential target. Trehalose is a simple nonreducing disaccharide containing two glucose units and plays critical roles in general stress adaptation in fungi, as well as energy reserves in certain fungi that might be used for ATP production under certain stresses [49–51]. Trehalose can interact with proteins and phospholipids to protect cell structures such as the plasma membrane from degradation, along with denaturation of intracellular proteins. This cell stress protectant has been shown to be important in fungal adaptation to mammalian body temperatures. In addition, studies with human pathogens suggest that trehalose is a reactive oxygen species scavenger and may protect against host oxidative burst [48]. During central nervous system infection, *C. neoformans* expresses high amounts of trehalose synthase 1 gene (*tps1*). Consistent with this result, cryptococcomas accumulates considerable quantities of this disaccharide. Moreover, functional studies with *C. neoformans* and *C. gattii tps1* mutants revealed that this gene is important for growth at 37°C. However, deletion of *tps1* has a fungicidal effect in host tissues. Thus, such evidence strongly indicates that trehalose counteracts host-imposed stresses other than temperature. This hypothesis was validated when cryptococcal ∆*tps1* was found to be severely attenuated in nematode and zebrafish models, in which temperatures fell below mammalian temperatures [52, 53]. TPS2 is a very specific phosphatase with no characteristics that suggest it as a promiscuous phosphate enzyme target. The trehalose phosphate synthase 2 is essential to keep *C. neoformans* growing at human body temperature; furthermore, its absence substantially impairs fungal virulence. This enzyme is a broad-spectrum target, as strains of *C. albicans* and *A. fumigatus* lacking its activity also exhibit decreased virulence. Trehalases degrade trehalose in glucose via a fuel-providing mechanism in fungi [54]. Although neutral trehalase genes do not have an impact on *C. albicans* and *C. neoformans*, an acidic enzyme influences *C. albicans* pathogenesis [55]. Validamycin A, a trehalase inhibitor, has a limited effect as an antifungal on *C. albicans* infection [56]. Thus, trehalases are still not potential targets due to the presence of multiple enzymes and different impacts on fungal cells. There is no data indicating them as a broad-spectrum target. Future efforts are encouraged to explore the impact of trehalose metabolism on fungal biology and virulence.

### 3.2. Amino Acid Metabolism-Related Targets

Amino acid biosynthetic routes have been shown as druggable targets, because during infection, fungi are exposed to nutritional stresses that require amino acid metabolism and present fungus-specific enzymes/processes. Evidence shows that efficient responses to amino acid starvation and requirements are important for fungal pathogenicity; the absence of CpcA, a transcriptional activator in amino acid starvation, and AreA, a nitrogen metabolic repressor activated when preferable nitrogen sources are not available, impairs *A. fumigatus* virulence in a murine model of pulmonary aspergillosis [57, 58]. Growing knowledge suggests that biosynthetic pathways have purposes beyond nutritional requirements; additional functions may exists that could be even more important for pathogenicity [59]. During fungal infection, the microorganism encounters specific niches with variable amounts of amino acids/nitrogen sources. Consequently, different infection models or routes may interfere in the essentiality of a specific amino acid biosynthesis in infection. *A. fumigatus* that lacks the HscA gene (homocitrate synthase) required for lysine biosynthesis revealed that spores, but not hyphae, need free lysine to grow, while hyphae use proteases to harvest lysine. Infection experiments additionally demonstrated that in inhalation and disseminated models, mutants presented avirulence and full virulence, respectively, indicating differential availability of lysine in these host niches [60]. More congruent niche-specific phenotypes were observed in isoleucine/valine auxotrophic *A. fumigatus* strain, which lacks dihydroxy-acid dehydratase (*ilv3A*); ∆*ilv3A* cells were avirulent in systemic infection but only slightly attenuated in pulmonary infection. Interestingly, the depletion of the paralogue *ilv3B* resulted in fungal avirulence in any infection model [61]. However, some biosynthetic pathways rise as niche-independent routes that are essential for fungal pathogenicity. The *A. fumigatus* HisB (imidazoleglycerol phosphates dehydrogenase) null mutant is unable to grow in blood agar and hydrolyzed BSA as a nitrogen source in vitro; thus, it is unable to establish infection in the lungs and bloodstream, which suggests that those niches are unable to provide sufficient amounts of histidine to support fungal growth.

Some amino acid biosynthetic genes are essential for fungi. For instance, *A. fumigatus* mutants that exhibit auxotrophy for the three aromatic amino acids are impossible to obtain. In addition, the *AroM* (dehydroquinate hydrolyase) mutant could not be generated because of its essentiality. As a result, a conditional promoter strategy driving the expression of A*roB* (chorismate synthase) was used to obtain aromatic amino acid auxotroph strain, which was unable to grow in medium containing the three aromatic amino acids and displayed attenuated virulence in pulmonary and systemic murine infection models. This fact could be explained by the accumulation of toxic chorismatic acid, a mitochondrial function inhibitor [62]. Tryptophan biosynthetic genes of *C. neoformans* seem to be essential for this fungus; however, exogenous supplementation with tryptophan, in nitrogen-depressed conditions (using proline as a nitrogen source), partially restored growth [63]. Conditional threonine mutants of *C. neoformans* were more susceptible to growth at human body temperature, but growth was partially rescued when threonine dipeptides were offered [64]. Although biosynthesis of amino acids has been explored in *A. fumigatus* and *C. neoformans*, knowledge of those pathways in dimorphic fungi such as *Blastomyces dermatitidis*, *H. capsulatum*, *Penicillium marneffei*, and *Paracoccidioides* is still poor. Based on our current knowledge, amino acid biosynthetic routes are suitable targets for antifungal development, because they are essential for fungal pathogens and have enzymes not found in humans. However, some aspects must be considered in targeting amino acid pathways: auxotrophy for certain amino acids can be restored by uptake of exogenous molecules from proteolytic products of fungal proteases and the niche-specific requirement varies dramatically [59]. Thus, the generation of conditional expressing mutants appears to be the best strategy to analyze in vivo amino acid acquisition, when null mutants are not obtainable.

### 3.3. Mitochondrial Proteins as Antifungal Targets

Comparison of mitochondrial genomes across databases resulted in evidence that some mitochondrial protein complexes may present species-specific proteins. Further, analysis of fungal mitochondrial proteins identified both conserved and fungus-specific molecules, among them were potential targets for the development of antifungal therapies. However, functional assays will be required to determine their role in pathogenesis. Current data from functional studies show a correlation of defects in mitochondrial function with virulence attenuation in *C. albicans*. The Ras1-Cyr-PKA signaling pathway controls fungal virulence and filamentation. The optimal functioning of this pathway requires a high cell energy status. The RAS pathway interacts with complexes I and IV but not with complex II or alternative oxidase [65]. Nuo1 and Nuo2 are NADH:ubiquinone oxidoreductase proteins that were identified as nonmammalian complex I proteins and are broadly conserved among fungi. The mutants for these molecules presented low ATP synthesis and respiration, defects in complex I assembly, and avirulence in a mouse model, which confirm these two proteins as interesting targets for antifungal development [66, 67]. Mitochondrial biogenesis includes the SAM (sorting and assembly machinery) and ERMES (ER-mitochondria encounter structure) protein complexes, which are crucial for the import of proteins into the intermembrane space and then to the matrix with joint action of the membrane transporters. The disruption of Sam57 or Mmm1, belonging to SAM and ERMES complexes, respectively, resulted in avirulent *C. albicans* strains [68, 69]. Another fungal-specific protein from the ERMES system is the GTPase Fzo. The lack of this protein is related to increased hydrogen peroxide and azole susceptibility, probably due to the energy-dependent drug efflux pumps [66]. Some studies have identified fission/fusion mitochondrially related genes in the pathogen *A. fumigatus*. The fusion genes *Mgm1*, *Ugo1*, and *Fzo1* play roles in fungal viability and virulence in a galleria model. In contrast, fission mutants showed impaired sporulation and are not essential for virulence [70]. A recent investigation identified a broad-spectrum antifungal candidate with activity against *Candida*, *Aspergillus*, and *Cryptococcus*, namely, ilicicolin. This natural polyketide inhibits the cytochrome Bc1 reductase of complex III and has no effect on the mammalian enzyme [71–73]. Thus, several fungal-specific mitochondrial proteins are promising because inhibition of these genes/proteins abrogates fungal pathogenicity.

### 3.4. Alternative Carbon Source Pathways

Carbon sources may vary and fungal nutritional requirement differs throughout the course and site of infection. In addition to the abovementioned amino acid-related attenuation of virulence, nonmammalian carbon metabolizing pathways have been described as important pieces of the fungal virulence arsenal. Transcription profile studies indicate that *C. albicans* downregulates glycolytic genes and upregulates glyoxylate cycle genes, which facilitate the assimilation of two carbon compounds with generation of anaplerotic oxaloacetate to gluconeogenesis. Further studies showed that yeasts use the glyoxylate cycle and gluconeogenesis when inside the phagosome, while the glycolysis supports survival in tissues [74–76]. However, other pathogenic fungi are not strictly dependent on the glyoxylate cycle for successful colonization; *A. fumigatus* strains tested for the impact of isocitrate lyase (ICL) resulted in no virulence attenuation [77]. Similar results for ICL mutants were observed in *C. neoformans* [78]. Differently, *A. fumigatus* relies more on the methylcitrate cycle (MCC) for survival in mammalian hosts [79]. Experimental infections with a methylcitrate synthase mutant showed that efficient degradation of propionyl-CoA is required for pathogenicity in a murine model of disease and, furthermore, the fungal cells were cleared from host tissues [80]. In this respect, transcriptional and proteomic analyses support the hypothesis that dimorphic fungi may rely on the MCC to adapt to carbon sources in host niches. The mRNA and protein of MCC-specific genes accumulate in infection-like conditions and during mycelium-to-yeast differentiation [81–85]. Additionally, *B. dermatitidis* cells recovered from mouse lungs show increased levels of mRNAs encoding MCC genes, indicating that fungal cells are exposed to propionyl-CoA-generating compounds [86].

### 3.5. Vitamin Synthesis, Ergosterol Metabolism, and Cell Wall as Targets for the Development of New Antifungals

Proteins involved in de novo vitamin biosynthesis are also suitable targets for antimycotic therapies. Unlike *C. albicans* and *A. fumigatus*, *H. capsulatum* is strongly dependent on phagocytes. This fungus is able to survive and grow inside phagosomes of the host's immune cells. *H. capsulatum* strains with interrupted biosynthesis of panthothenate and riboflavin were unable to proliferate in macrophage phagosomes and revealed attenuated virulence in vivo [87]. Thus, as these routes compose the set of metabolic adaptation to host conditions and are absent in humans, their components are strong candidates for antifungal therapy.

The metabolic processes of ergosterol biosynthesis, membrane stability and maintenance, cell wall remodeling, and folate synthesis are long-established drug targets. Although the existing antifungal agents used to inhibit the abovementioned targets exhibit toxicity for patients and resistant strains are often identified, these pathways are still considered to be in the pipeline for antifungal drug development, because they are absent in mammals and important for virulence. Polyoxins are peptide-derived compounds from *Streptomyces* that inhibit chitin synthesis and consequently are a promising therapy option. Drug repositioning and rational design strategies are used to elaborate new fungicidal compounds. The azole class of antifungals is a great option to manage mycoses; nonetheless, drugs that stick to heme-binding site of *Erg11* also target host cytochromes, resulting in liver toxicity. Recently, the compound VT-1161, a new azole, was rationally designed to have low affinity to mammalian proteins and high efficiency against *C. albicans* and *Coccidioides* [88, 89]. This promising antifungal has recently entered clinical trials [90]. Repositioning of anticancer drugs rendered the identification of AR-12, an antimicrobial compound that is highly potent against fungi. AR-12 inhibits acetyl-CoA synthetase, an essential fungal enzyme [90], and is effective against a broad collection within the kingdom of fungi, including the dimorphic clade [91].

## 4. Strategies to Develop New Antifungal Agents

Fungal infections represent an important medical issue particularly for immunocompromised patients, such as those with organ transplants or suffering from cancer and HIV infection, for whom fungal infections are frequently life-threatening [92–96]. The rate of these fungal infections has increased significantly in recent decades; pathogenic fungi are responsible for approximately 1.5 million cases of infection per year [92–96]. Despite these alarming statistics, the impact of fungal infections on human health has generally been neglected [97].

Although drugs with antifungal properties are available, they are limited when compared to antibacterial drugs. While the discovery of drugs based on polyenes, azoles, and allylamines may represent significant advances in the field of antifungal agent research, several challenges common to other pathogenic organisms such as side effects, narrow spectrum of activity, and the development of drug-resistant fungi must be overcome [98, 99]. The majority of antifungal drugs, except for amphotericin B, have a fungistatic effect [100]. However, the use of amphotericin B is restricted due to its side effects [100, 101]. Moreover, fungi are eukaryotic parasites that colonize a eukaryotic host, and the narrow range of physiologic differences between the host and colonizer may represent hurdles to developing safe and wide-ranging antifungal agents.

Several strategies may be employed to develop drugs; one of the foremost requirements for drug development is identification of relevant cellular targets to test these therapies. In this regard, identification and development of novel antifungal agents that have minimal toxicity to the host, as well as identification of fungal-specific molecular targets, are essential. Notably, distinct strategies can be applied to develop new antifungal agents to circumvent fungal resistance or promote a better quality of life for patients affected by fungal infections. One of the most employed methods for the development of new therapeuticals involves identifying bioactive compounds present in plants, animals, and microorganisms [99, 102–106]. In addition, a recent approach applies bioinformatics analysis to search in genomic databases for peptide sequences that have the physical-chemical characteristics of the antifungal drugs [107–109]. This may employ the use of several methodologies, including global approaches such as transcriptomic and proteomic methods that may contribute to the understanding of the mechanism of action of these new drugs [110, 111]. The next sections describe different approaches that could lead to the development of new antifungal compounds.

### 4.1. Identification of Bioactive Compounds

Natural compounds are widely used to identify antifungal molecules, and several natural compounds from various classes such as essential oils [112], lignan (reviewed in [113]), and curcumin (reviewed in [114]) have been studied.

Screening of libraries of synthetic small molecules or natural products is one of the most employed methods to identify drugs [99, 102, 103] and represents the vast majority of the available antibiotics for clinical use [99, 115–117].

Studies performed by Shi and colleagues characterized two acetophenone derivatives, 2-hydroxy-4,6-dimethoxyacetophenone and 2,4-dihydroxy-5-methylacetophenon, as plant antifungal agents [118]. These compounds were isolated from *Melicope borbonica* and *Polyporus picipes*, respectively, and were demonstrated to be effective in *Cytospora* sp., *Glomerella cingulata*, *Pyricularia oryzae*, *Botrytis cinerea*, and *Alternaria solani* [118]. Therefore, this strategy may be employed to screen for antifungal agents against human pathogenic fungi.

In fact, the antifungal agents, echinocandins and polyenes, were discovered through screening of natural products [119–121], and recently, Hein and colleagues reported the isolation of psoriasin from lesional psoriasis scale extracts. They identified this molecule as a potent fungicidal protein active against *Trichophyton rubrum* and *A. fumigatus*. The proposed mechanism of action is chelation of free intracellular zinc, which leads to fungal apoptosis [122]. It is important to note that biofilms are important in clinical settings mainly because they are associated with high drug resistance [123–126]. Recently, Seleem et al. demonstrated the effect of lichochalcone-A, a natural compound found in licorice roots of *Glycyrrhiza* species, on biofilms produced by *C. albicans* [127]. In this work, the authors showed that lichochalcone-A reduces *C. albicans* biofilm and decreases the proteolytic enzymatic activities of proteinases and phospholipases secreted by this fungus. Most importantly, mice treated with lichochalcone-A exhibited a significant reduction in fungal load five days postinfection, suggesting that this compound may be a candidate for a preclinical trial [127].

Despite the promise of these strategies, a problem encountered in the use of these molecules, which is in the majority of peptides, is their poor stability when applied in vivo. Peptides belonging to the most bioactive compounds were explored as antifungal agents. They can be obtained as metabolites of plants, insects, animals, and microorganisms [104, 105, 107] as the first-line arsenal to combat infections. These molecules are attracted to act against fungi because of a conformational structure that generally interacts with the fungal membrane causing an imbalance in the cell.

In general, the ability of antimicrobial peptides (AMPs) to destabilize microbial membranes is due to the conformation of these molecules that are approximately 50 amino acid residues long, with an overall residual positive charge and hydrophobic residues that provide an amphiphilic three-dimensional structure [107, 128]. Unlike most classic antifungal agents, their mechanism of action involves interruption of ergosterol biosynthesis. AMPs directly disrupt membrane integrity, culminating in cell death. Experimental data suggest that the mechanism of action of peptide-like lactoferrin B (Lfcin B) on biological membranes (using liposomes as a membrane model) is the formation of pores that results in loss of cell components [129].

Two cathelicidins (a LL-37 linear alpha-helical cationic peptide identified in the human vaginal tract and the bovine ortholog BMAP-28 peptide) were evaluated in vitro against *Candida* spp. isolated from patients. The mechanism of action for cathelicidins is to target the cell membrane, which causes its disruption and release of the intracellular components [125, 130]. They showed equal or better activity against biofilms formed by *Candida albicans* SC5314 compared to the antifungal agents, miconazole and amphotericin B (AMB). It was also observed that pH influenced the bioactivity of these peptides [125].

Oguro and colleagues demonstrated that a defensin (which is approximately 50 amino acid residues long and is frequently rich in cysteine) from *Brassica juncea* is capable of causing permeabilization and production of reactive oxygen species (ROS) in *C. albicans* by binding to sphingolipid glucosylceramide [131]. Generally, these molecules interact with membranes, inducing oxidative damage by an excessive increase in ROS [132].

The histatins are another group of amphipathic AMPs with helical structure that is important for their antifungal activity. Histatin 5 is found in human saliva and is toxic to fungal cells but causes low toxicity to human cells. It binds to fungal cell wall proteins, causing the release of intracellular ATP-activating P2X receptors in membranes forming ion channels [49, 50]. Because this molecule is capable of preventing biofilm formation caused by *C. albicans* [133], its hybridization with other molecules [134] has been used to improve the effect of these AMPs, which could possibly be applied in formulations to treat oropharyngeal candidiasis.

Despite the antimicrobial potential of these classes of biomolecules, they may be unstable when in biological media. For example, when exposed to pH values, which are not ideal for a structural conformation that presents antifungal activity, they may no longer be efficient. In addition, the histatins are amphipathic AMPs with helical structure that is important for their antifungal activity. Microorganisms can induce the production of metabolites by the host affecting AMP activity [135]. *C. albicans* can degrade LL-37 peptide by expressing secreted aspartic protease (SAP) family [136].

While these AMPs identified in natural sources are good candidates to be used in the development of new antifungal drugs, they must be chemically synthesized with various changes in their structure to improve their activity. López-Abarrategui et al. [106] presented a good example of changes in cationicity and hydrophobicity properties and Boman index in a peptide extracted from a mollusk. In this study, a more than three-fold increase in AMP activity against *C. albicans* was observed, compared to that in the wild-type peptide [106].

Based on the properties described for AMP, computational programs have been developed to identify AMP-similar sequences by exploring the genomic databases of sequenced organisms. Amaral et al. reported the identification of four peptide sequences, two in the human genome and two in the *Paracoccidioides brasiliensis* transcriptome, which are potential candidates as antifungal agents [107].

However, peptides often do not present the expected activity in vivo even when they present good in vitro results for several reasons, including enzymatic degradation or peptide structure destabilization [137]. A possible alternative to solve these issues is by incorporating these molecules into nanostructured drug delivery systems [138]. Such systems are prepared using nanotechnological approaches that allow rational delivery of bioactive compounds, such as AMP.

### 4.2. Nanostructured Antifungals

According to some studies, nanotechnology is capable of improving antifungal activity for both conventional drugs [139] and bioactive molecules [140]. These improvements may be attributed to nanoscale properties that allow biomolecule protection against biodegradation. Association of AMP with a nanoparticle-based drug delivery system can increase its delivery to the site of action.

d'Angelo et al. developed a nanostructured system for the delivery of colistin, a cationic AMP within poly(lactic-*co*-glycolic acid) (PLGA) nanoparticles engineered with mucoadhesive chitosan [137]. In addition to allowing the protection of peptides against degradation, this approach also permits more effective targeting to the site of action.

Nanotechnology has also been applied to exploit the antimicrobial properties of certain nanomaterials such as silver nanoparticles, which are naturally effective against microorganisms by destabilizing their membrane through electrostatic interactions [138]. However, these nanoparticles also exhibit high toxicity to mammalian cells, which can compromise their use. One way to avoid or reduce this undesirable effect is by incorporating AMP onto the surface of these nanoparticles, decreasing the toxicity against erythrocytes [141]. This combination increased AMP efficiency and further increased its stability compared to the AMP without complexation with nanoparticles. Silver nanoparticles coated with a peptide had diminished cytotoxicity against erythrocytes [141].

Nanotechnology properties are also used to enhance the activity of classic antifungals, especially when the aim is to reduce their unwanted toxic effects, such as in amphotericin B (AMB), which is considered one of the front lines in antifungal therapy [139, 142]. A classic nanostructured formulation for AMB is AmBisome®, a liposomal formulation that minimizes unwanted toxic effects of this potential drug.

Amaral et al. developed a nanoformulation containing AMB within PLGA and functionalized with dimercaptosuccinic acid (DMSA), which presents a tropism to the lungs. The formulation showed no genotoxicity and its therapeutic effect was better than that observed in vivo for AMB deoxycholate in a chronic paracoccidioidomycosis murine model [139]. The authors suggested that the effect might be due to DMSA, which drives the entire nanostructure toward the lungs, guiding the drug to the site of action.

Xu and colleagues also tested another polymeric formulation for AMB using *α*-butyl-cyanoacrylate in experimental meningitis caused by *C. neoformans* [143]. In this study, the AMB in brain tissue was detected 0.5 hours after injection into animals and a maximum AMB concentration was detected within 3 hours when compared to that in the animals treated with conventional AMB. In addition, animals with cryptococcal meningitis treated with the formulation showed a high survival rate [143]. This nanoformulation was first developed by the same research group that demonstrated the potential of nanoparticles containing AMB coated with polysorbate 80 to deliver this drug across blood brain barrier when compared to the conventional formulation for AMB [144]. Thus, nanoformulations could be used to deliver toxic drugs, such as AMB, in cases of antimicrobial resistance to less toxic drugs, such as azoles, used mainly by immunocompromised patients.

### 4.3. Drug Repositioning

Although the approaches described above are important for antifungal drug development, it is important to emphasize that the process of drug discovery and development requires 10–17 years on average and the success rate is lower than 10%. A repositioning approach is based on previous research and development of conventional medications that have already been tested in humans in terms of toxicology and pharmacology [145–149]. In this context, drug-repositioning screening may be a valid and affordable alternative method to obtain antifungal drugs. In fact, this strategy has been employed to expedite identification of new therapeutic applications and has been successfully used in therapy of parasitic diseases [150] and several types of cancer [151, 152].

Sun and colleagues, using high-throughput assays, screened approved drugs to identify potent effects against *Exserohilum rostratum* infections [153]. They identified bithionol (antiparasitic drug), tacrolimus (immunosuppressive agent), and floxuridine (antimetabolite) as anti-*E. rostratum* agents, confirming that drug repositioning can be performed [153].

Another approach using repositioning drugs as antifungal agents has been performed. In addition to identifying a drug that has an antifungal effect individually, researchers are working to identify compounds that may synergize with available antifungal drugs. An outstanding example of this approach was performed by Robbins et al. [154]. In this work, a library of compounds was evaluated in combination with subinhibitory concentrations of known antifungals such as amphotericin B, fluconazole, terbinafine, caspofungin, benomyl, and cyprodinil. Remarkably, this approach led to the identification of a synergistic effect with clofazimine (an antimycobacterial drug not reported as antifungal) with caspofungin and posaconazole, indicating that this may represent a potential therapeutic approach against diverse fungal pathogens.

## 5. Vaccine Development against Fungal Diseases

The immune response against microorganisms such as fungi consists of two major systems: the innate and adaptive immune responses. The adaptive immune response is constituted by cellular and humoral immune responses. The innate response is the first pathway for fungal detection. Immune cells recognize fungal pathogen-associated molecular patterns (PAMP) through pattern recognition receptors (PRR) present in host cells. After invasion by pathogens and recognition, cells are recruited and stimulated to the infection site, such as monocytes, neutrophils, macrophages, natural killer cells, and dendritic cells, which develop an important role to link and stimulate the adaptive immune response (reviewed in [155, 156]).

When activated, those cells secrete soluble molecules that participate in effector mechanisms for fungal clearance. Among these molecules, there are complement proteins, antimicrobial peptides, costimulatory molecules, chemokines, and cytokines. Phagocytic cells are sufficient to trigger all effector mechanisms. Phagocytosis initiates a signaling pathway, and this microenvironment determines the cytokine pattern and antimicrobial functions that will be developed. Both innate and adaptive immune responses will work together. Furthermore, the effective mechanism to adaptive immune response is based on innate response (reviewed in [155, 156]).

There are different classes of cytokines as proinflammatory (IL-1, IL-6, IL-17, IFN-*γ*, and TNF-*α*), anti-inflammatory (IL-4, IL-13, and TGF-*β*), and immune regulatory (IL-10). When proinflammatory cytokines are prevalent, cellular immunity mediated by CD4^+^ T cells (Th1 and Th17) is stimulated, whereas when these cytokines are downregulated, humoral immunity takes place (CD4^+^ T cells—Th2, or regulatory T cells (Treg)). Both types of cytokines stimulate antibody production, fungal killing, transcription factors such as NF-*κ*B. It is widely accepted that induction of Th1-/Th17-type cellular response is crucial for the defense against fungal infection. Th2 humoral response is usually considered not protector, since it stimulates antibody switching to nonopsonizing isotypes. The isotype IgG2a is considered the most protective antibody for fungal infection because it induces a cross-talking with effector molecules of cellular response. Cytotoxic T cells (CD8^+^ T cells) are the major producers of IFN-*γ*, TNF-*α*, and IL-2. These cells naturally occur in the host response to fungal pathogens in the lungs. In conclusion, the interaction of fungal derivatives with immune cells depends on the nature of compounds for yeasts/filamentous and is developed by the complex microenvironment (reviewed in [155, 156]).

The use of vaccine strategies that can be applied together with drug therapy could reduce treatment times, reestablish a protective immune response, and prevent posttreatment sequelae [157]. In the past decade, researchers have been investigating vaccine development against the major human and veterinary fungal pathogens. Most studies are focused on protection against the pathogenic fungi *Aspergillus* spp. [158], *Candida* spp. [159], *B. dermatitidis* [160], *Cryptococcus* spp. [161], *Coccidioides* spp., *Histoplasma capsulatum* [162], *Microsporum canis* [163], *Paracoccidioides* spp. [164], *Pneumocystis jirovecii* [165], *Sporothrix* spp. [166], and *Talaromyces* (*Penicillium*) *marneffei* [167, 168], which cause cutaneous, subcutaneous, and systemic mycoses in all regions of the world and account for millions of new infections per year. The development of safe and efficacious vaccines against fungi is still a challenge due to our lack of knowledge about immunity against fungal infection. However, the use of animal models is helping us to better understand the interaction between the immune system and fungi [169].

There is no doubt that CD4^+^ T cells play a major role in mediating resistance against fungal infection in immunocompetent and immunosuppressed patients [170]. The mechanism of resistance conferred by CD4^+^ T cells occurs through secretion of T-helper 1 (Th1) or Th17 cytokines such as IFN-*γ*, TNF-*α*, GM-CSF, and IL-17A, which activate various cell populations including neutrophils, macrophages, and dendritic cells. Natural killer cells and natural killer T cells also play an important role in fungal control by modulating immune response through the production of proinflammatory cytokines such as IFN-*γ* and TNF-*α* [171–174]. The function of B cells and production of protective antibodies were once controversial. However, today, it is known that the protective function of antibodies depends on various factors [175–177]. Although CD4^+^ T cells play a major role in fungal control, mediation of protective immunity by CD8^+^ T cells against fungal infection has been documented. The studies involving CD8^+^ T cells as vaccine strategies gain special importance in patients with impaired CD4^+^ T cells as occurring under HIV infection [169, 178].

Protective immunity mediated by CD8^+^ T cells has been documented in different fungal infections such as aspergillosis, histoplasmosis, cryptococcosis, blastomycosis, paracoccidioidomycosis, pnemocystosis, and mucosal candidiasis. Usually, antifungal CD8^+^ T cells are elicited by cross-presentation of fungal peptides by MHC class I. Antifungal immunity in the absence of the CD4^+^ T cell can be mediated by cytotoxic type I CD8^+^ T cells, which secrete IFN-*γ*, TNF-*α*, and GM-CSF, as well as by the cytotoxic factors perforin, granulysin, and granzyme K or IL-17/IFN-*γ* double-producing CD8^+^ T cells [169]. Nanjappa et al. showed that even in the absence of CD4^+^ T cells, vaccinated mice with 105-106 yeast of attenuated *B. dermatitidis* induced IL-17 producing CD8^+^ T cell, which conferred resistance against *B. dermatitidis* infection [179]. Although there is no licensed vaccine for humans, the results of experimental models are promising and can indicate candidates for clinical trials. Below, we will discuss some vaccine models in development against endemic fungal infections.

### 5.1. *P. brasiliensis* and *P. lutzii*

A main candidate for a vaccine against *P. brasiliensis* is the P10 peptide, whose sequence is QTLIAIHTLAIRYAN. CD4^+^ T cell modulation with a significant increase in IL-12 and IFN-*γ* and a decrease in IL-4 and IL-10 was observed in mice immunized with P10 alone or in association with antifungal drugs [180]. Another antigenic protein from *P. brasiliensis*, rPb27, showed protective results in an experimental model using mice [181]. The immunization of BALB/c mice with radioattenuated yeast cells of *P. brasiliensis* promoted long-lasting protection against an infective yeast form [182]. The passive transfer of monoclonal antibodies against the 43 kDa glycoprotein gp43 [183] or gp70 [184] from *P. brasiliensis* was shown to be protective against experimental infection of *P. brasiliensis*. Monoclonal antibodies generated against the heat shock protein 60 from *H. capsulatum* also interact with *P. lutzii* yeast cells and enhance phagocytosis by macrophage cells. The passive transfer of 7B6 and 4E12 mAbs against Hsp60 was protective and reduced the fungal burden in the lungs of BALB/c mice intratracheally infected with *P. lutzii* [175].

### 5.2. *H. capsulatum*

Vaccination with recombinant HSP60 from *H. capsulatum* is able to elicit protection mediated by CD4^+^ T cells and induces IFN-*γ* production [185]. Immunization with apoptotic phagocytes containing heat-killed *H. capsulatum* efficiently activated CD8^+^ T cells, whose contribution was equal to that of CD4^+^ T cells in protecting against *Histoplasma* challenge [186]. Treatment of mice with a monoclonal antibody against HSP60 before infection reduced the fungal burden in the lungs [175].

### 5.3. *Coccidioides posadasii* and *C. immitis*

The first vaccine tested against endemic mycosis using a killed spherule vaccine failed during a phase 3 clinical trial [169]. A decapeptide agonist of the biologically active C-terminal region of the human complement component C5a, termed EP67, was conjugated with lysine residues on the surface of live arthroconidia. The use of the EP67 vaccine induced phagocytosis and antigen presentation. BALB/c mice immunized with conjugated EP67 increased survival and reduced inflammatory pathology and fungal burden. This protection was mediated by augmenting the T-helper 1 (Th1) and Th17 responses [187, 188]. Whole glucan particles prepared from *Saccharomyces cerevisiae* conjugated with BSA induced significant protection in CD-1 mice [189]. A recombinant Ag2/PRA106 + CSA chimeric fusion protein vaccine in ISS/Montanide adjuvant administered intramuscularly showed promising results in adult female cynomolgus macaques challenged with *C. posadasii* [190]. Synthetic peptides corresponding to the five selected epitopes from fungal aspartyl protease, alpha-mannosidase, and phospholipidase B were incorporated into the vaccine with synthesized CpG ODN in incomplete Freund's adjuvant. Mice vaccinated and challenged intranasally with *C. posadasii* showed reduced fungal burden and infiltration of activated T-helper 1 (Th1), Th2, and Th17 cells, as well as elevated IFN-*γ* and IL-17 [2].

### 5.4. *Sporothrix brasiliensis* and *S. schenckii*

The passive transfer of monoclonal antibody P6E7 (which recognizes a 70 kDa glycoprotein—gp70—an important factor of virulence) reduced the fungal burden of mice infected with some but not all *Sporothrix* isolates tested. Protection was detected during the early stages and relapse in the final period of infection (21 days) [191].

Serum from mice immunized with a 44 kDa (peptide hydrolase) and 47 kDa (enolase) *Sporothrix schenckii* cell wall protein (predicted to be an adhesein) in aluminum hydroxide was used in a passive transfer to mice systemically infected with *S. schenckii* and resulted in protection. In vitro assays with yeast cells opsonized with serum from immunized animals increased phagocytosis and inhibited the adhesion of the fungi to the fibroblasts [192].

### 5.5. *B. dermatitidis*

Virulence-attenuated yeast cells of *B. dermatitidis* (strain 55) were injected subcutaneously into C57Bl/6 mice. Animals were challenged by intratracheal infection with wild-type *B. dermatitidis* 26199 yeasts. Vaccine-induced protection requires dectin-2 to promote differentiation of activated T-helper 1 (Th1) and Th17 cells [160]. A genetically engineered live-attenuated strain of *B. dermatitidis* lacking the major virulence factor BAD-1 was shown to be protective against lethal experimental infection in mice [193]; vaccine immunity in endemic dimorphic fungal infection is primarily mediated by T-helper 1 (Th1) and Th17 cells, but not by antibodies as observed in the experimental model using mice [194]. The use of BAD-1-deficient *B. dermatitidis* has also been investigated as a pan-fungal vaccine against endemic mycosis in North America (*C. posadasii*, *H. capsulatum*, and *B. dermatitidis*). This vaccine induced Th17 and was sufficient to protect against all fungi tested. The protection was mediated by Th17 cells, which recruited and activated neutrophils and macrophages to the alveolar space [162].

### 5.6. *Aspergillus* spp. and *Candida* spp

There are no licensed vaccines against *Aspergillus*. The literature described four *Aspergillus* vaccine categories: pan-fungal, crude, subunit, and therapeutic. All candidates for *Aspergillus* vaccines are being tested in an experimental mice model. A pan-fungal vaccine has advantages when compared with other categories of vaccines (reviewed in [195]). A conjugated *β*-1,3-D-glucan to diphtheria toxin has been shown to be immunogenic, and antibodies were protective as an immunoprophylactic vaccine against systemic and vaginal *Candida albicans* and *A. fumigatus* [196]. On the other hand, subcutaneous mice vaccination with heat-killed *Saccharomyces cerevisiae* yeast protected against *Aspergillus*, *Coccidioides*, or *Candida* challenge [158]. Using transgenic CD4^+^ T cells, a sequence of amino acids was identified within chaperone calnexin that is conserved in phylum Ascomycota. Vaccine-conjugated recombinant calnexin protein or calnexin peptide with different adjuvants induced resistance to lethal challenge against *B. dermatitidis*, *H. capsulatum*, and *C. posadasii*. Although this vaccine has not been tested against *Aspergillus* and *Candida*, proteomic studies found potential [197]. The other categories, such as live or killed *A. fumigatus* (crude); recombinant proteins Asp f3, Gel1, Asp f9 (Crf1), Asp f16, and Pep1(subunit); or adoptive transfer of *Aspergillus*-specific CD4^+^ T cells (therapeutic), showed to be potential candidates for a human vaccine [195]. Due to the importance of both *Candida* spp. and *Aspergillus* spp. infections, there are several vaccine formulations in development. The most common protocols include the following: live-attenuated *C. albicans* strain, purified protein (Sap2p and Als3p), HSP90p, Hyr1p (glycosylphosphatidylinositol- (GPI-) anchored mannoprotein on the cell wall), cell wall extract (*β*-mercaptoethanol extract), glycoconjugated vaccines (polysaccharide as a carrier), *β*-mannan and peptide conjugates, and *β*-glucan conjugate with MF59 adjuvant. Although most experiments reported protection in murine models, there is no knowledge yet if these vaccines will be effective for human protection [198].

## 6. Concluding Remarks

It is possible that fungal infection will continue to rise as a result of increased numbers of immunocompromised patients. The development of new tools to treat patients with fungal infection is a priority. In addition to the discovery of new antifungal drugs, vaccines are an important alternative to be used alone or in combination with antifungal drugs. Researchers and the pharmaceutical industry are currently investing in the development of a pan-fungal vaccine to reach the largest number of patients.
